# Novel Histone Deacetylase Inhibitors and HIV-1 Latency-Reversing Agents Identified by Large-Scale Virtual Screening

**DOI:** 10.3389/fphar.2020.00905

**Published:** 2020-06-17

**Authors:** Donya Naz Divsalar, Conrad Veranso Simoben, Cole Schonhofer, Khumoekae Richard, Wolfgang Sippl, Fidele Ntie-Kang, Ian Tietjen

**Affiliations:** ^1^Faculty of Health Sciences, Simon Fraser University, Burnaby, BC, Canada; ^2^Department of Chemistry, University of Buea, Buea, Cameroon; ^3^Instutite of Pharmacy, Martin-Luther University Halle-Wittenberg, Halle (Saale), Germany; ^4^The Wistar Institute, Philadelphia, PA, United States

**Keywords:** virtual screening, histone deacetylase, histone deacetylase inhibitor, HIV, latency reversal, drug discovery

## Abstract

Current antiretroviral therapies used for HIV management do not target latent viral reservoirs in humans. The experimental “shock-and-kill” therapeutic approach involves use of latency-reversal agents (LRAs) that reactivate HIV expression in reservoir-containing cells, followed by infected cell elimination through viral or host immune cytopathic effects. Several LRAs that function as histone deacetylase (HDAC) inhibitors are reported to reverse HIV latency in cells and in clinical trials; however, none to date have consistently reduced viral reservoirs in humans, prompting a need to identify new LRAs. Toward this goal, we describe here a virtual screening (VS) approach which uses 14 reported HDAC inhibitors to probe PubChem and identifies 60 LRA candidates. We then show that four screening “hits” including (S)-*N*-Hydroxy-4-(3-methyl-2-phenylbutanamido)benzamide (compound **15**), *N*-(4-Aminophenyl)heptanamide (**16**), *N*-[4-(Heptanoylamino)phenyl]heptanamide (**17**), and 4-(1,3-Dioxo-1H-benzo[de]isoquinolin-2(3H)-yl)-*N*-(2-hydroxyethyl)butanamide (**18**) inhibit HDAC activity and/or reverse HIV latency *in vitro*. This study demonstrates and supports that VS-based approaches can readily identify novel HDAC inhibitors and LRAs, which in turn may help toward inhibitor design and chemical optimization efforts for improved HIV shock-and-kill-based efforts.

## Introduction

While combination antiretroviral therapy (cART) can durably suppress HIV replication, it does not act on resting CD4+ T cells containing latent proviral reservoirs. As these cells can reactivate at any time to produce infectious virus, they preclude a readily accessible HIV cure (Finzi et al., 1997; Siliciano et al., 2003; Cary et al., 2016). As a result, cART currently must be taken for life.

One proposed therapeutic-based method toward identifying and eliminating HIV reservoir-containing cells, frequently termed “shock-and-kill,” involves the use of latency reversal agents (LRAs) to initially stimulate virus production (i.e., “shock”). Following LRA administration, cells expressing reactivated HIV are then eliminated through apoptosis or immune-enhancing mechanisms (“kill”), while the concurrent use of cART prevents reservoir reseeding (Deeks, 2012). Among numerous LRAs identified to date, one of the most common functional classes is comprised of inhibitors of class I histone deacetylases (HDAC; Margolis, 2011; Zaikos et al., 2018). During proviral latency, HDACs are recruited to the HIV promoter resulting in transcriptional repression (Archin and Margolis, 2014; Darcis et al., 2017). In the presence of HDAC inhibitors, lysine acetylation within histone tails results in euchromatin formation and subsequent binding of transcription factors that drive provirus expression. Several HDAC inhibitors, including both natural products (e.g., romidepsin) and synthetic derivatives of natural product leads (e.g., vorinostat), have been investigated in clinical trials for their ability to reactivate provirus expression and reduce HIV reservoir levels in cART-treated, virally suppressed, HIV-infected individuals (Rasmussen and Lewin, 2016; Andersen et al., 2018). However, while a subset of these studies reports transient increases of virus or viral RNA following HDAC inhibitor administration, no trial to date has shown a significant reduction of HIV reservoir size in humans (Abner and Jordan, 2019; Zerbato et al., 2019). These results suggest that additional HDAC inhibitors with improved efficacy and/or selectivity for proviral integration sites may be needed to achieve sufficient HIV latency reversal and viral reservoir clearance *in vivo*.

One approach toward identifying new HDAC inhibitor prototypes, to serve as the basis for developing potentially improved efficacy and selectivity over existing agents, involves virtual screening (VS) of compound library databases. This method frequently entails searching for potential “hit” molecules which are stored in an electronic format and are able to interact favorably with a drug target site or otherwise “fit” into the structural, electronic, and steric features of known bioactive molecules *in silico*. Common VS approaches involve molecular docking of an electronic three dimensional molecular library toward a drug target site, searching for common pharmacophores, and calculating binding free energies (Chaput et al., 2016; Chen et al., 2017). Previous efforts by us and others have shown that “hits” identified from VS are frequently biologically active, for example as novel inhibitors of HIV replication (Brigo et al., 2005; Shityakov and Dandekar, 2010; Tietjen et al., 2015; Panwar and Singh, 2017; Berinyuy and Soliman, 2017). We therefore hypothesized that similar efforts could be used to identify novel LRA candidates.

Toward this goal, we describe here the results of a VS beginning with 5,687 unique compound structures obtained from ~100 million compounds present in PubChem (Kim et al., 2016). We describe a designed procedure that combines similarity searching, docking, and scoring and which resulted in the identification of 60 hit compounds as potential HDAC inhibitors. We then confirm that four commercially available compounds from this list function as HDAC inhibitors and/or HIV LRAs *in vitro*.

## Materials and Methods

### Reagents

Jurkat T cells (Clone E6-1) were obtained from the American Tissue Culture Collection. J-Lat 10.6 cells were obtained from the NIH AIDS Reagent Program, Division of AIDS, NIAID, NIH (contributed by Dr. Eric Verdin; Jordan et al., 2003). Cells were cultured in R10+ media [RPMI 1640 with HEPES and L-Glutamine, 10% fetal bovine serum, 100 U of penicillin/ml, and 100 µg of streptomycin/ml (Sigma)] and incubated at 37°C and 5% CO_2_.

HDAC-Glo I/II Assay kits were obtained from Promega. Panobinostat was obtained from Sigma-Aldrich. Compounds **15**, **16** and **17**, and **18** were purchased from Sellick Chemical, Enamine, and Molport, respectively. Serpulanine A was gratefully obtained as a gift from Dr. Raymond J. Andersen (University of British Columbia). Compounds stocks were diluted in DMSO and stored at minus 20°C until use.

### Dataset Collection

The VS dataset was collected from selected molecules in PubChem (Kim et al., 2016). We then searched for compounds from PubChem having close similarity with 14 known class I HDAC inhibitors including belinostat, entinostat, givinostat, mocetinostat, oxamflatin, panobinostat, psammaplin A, romidepsin, scriptaid, serpulanine A, thiophenyl benzamide, trichostatin A, valproic acid, and vorinostat (Kiernan et al., 1999; Contreras et al., 2009; Matalon et al., 2010; Ying et al., 2010; Matalon et al., 2011; Yin et al., 2011; Archin et al., 2012; Rasmussen et al., 2014; Wei et al., 2014; Huang et al., 2018; Richard et al., 2018; Williams et al., 2018; Zaikos et al., 2018; **Table 1**). A two dimensional (2D) similarity search was conducted on the PubChem website, with similarity between chemical structures being quantified by use of the Tanimoto equation (Chen and Reynolds, 2002; Holliday et al., 2002; Holliday et al., 2003) in combination with the PubChem substructure fingerprint (ftp://ftp.ncbi.nlm.nih.gov/pubchem/specifications/pubchem_fingerprints.pdf). For 12 of 14 compounds which were listed in PubChem at the time of study [all except the thiophenylbenzamide (compound **1**) and serpulanine A (compound **11**)], the canonical SMILES were used to conduct similarity searches. For the remaining two compounds (**1** and **11**), 2D structure files built with ChemDraw (.cdx) were converted to their respective.mol files, uploaded to PubChem, and used for the similarity search. Initially, a random search was carried out for 80%, 90%, and 95% Tanimoto similarities. The hit lists for similarity searches with less than 95% similarity cut offs yielded several hundred thousand entries for some of the 14 HDAC inhibitors; as a result, we selected a cut off of 95% similarity for all 14 input compounds. Resulting outputs from the search were collected into a combined dataset and duplicates were removed, leading to an initial library of 5,867 unique compound entries.

**Table 1:** Structures of reported histone deacetylase (HDAC) inhibitors used for virtual screening (VS).

Number	Compound	Structure	PubChem CID	Reference	N PubChem compounds with > 95% similarity
1	4-acetamido-*N*-(2-amino-5-thiophen-2-ylphenyl)benzamide	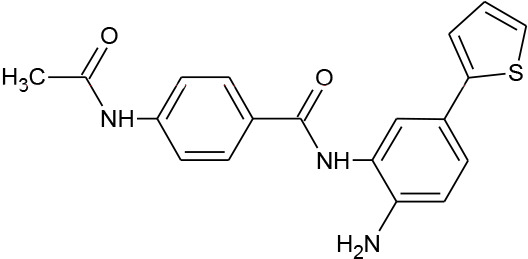	6918878	Huang et al., 2018	1,365
2	Belinostat	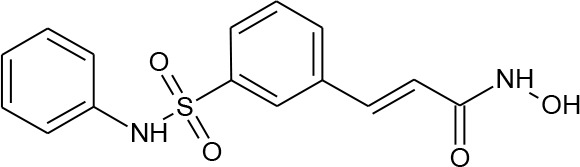	6918638	Matalon et al., 2011	169
3	Etinostat	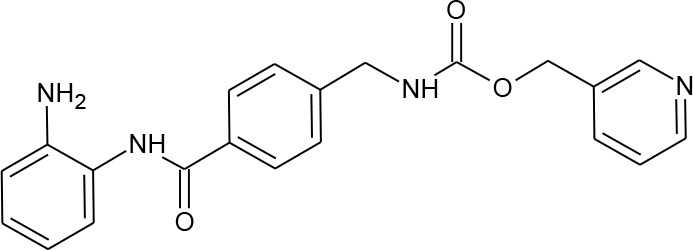	4261	Zaikos et al., 2018	213
4	Givinostat	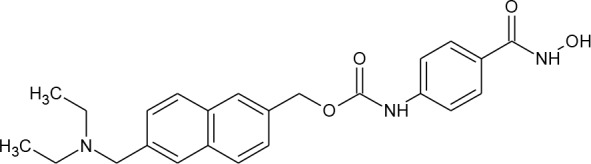	9804992	Matalon et al., 2010	37
5	Mocetinostat	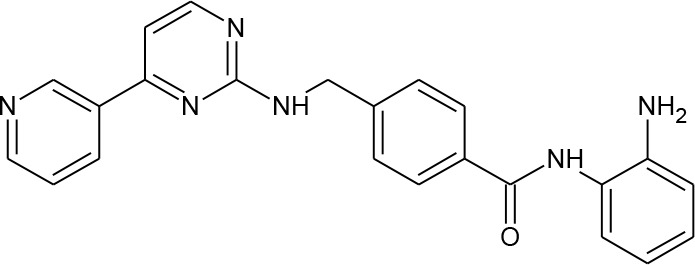	9865515	Zaikos et al., 2018	1,645
6	Oxamflatin	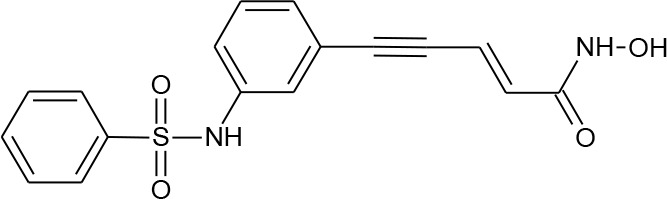	5353852	Yin et al., 2011	114
7	Panobinostat	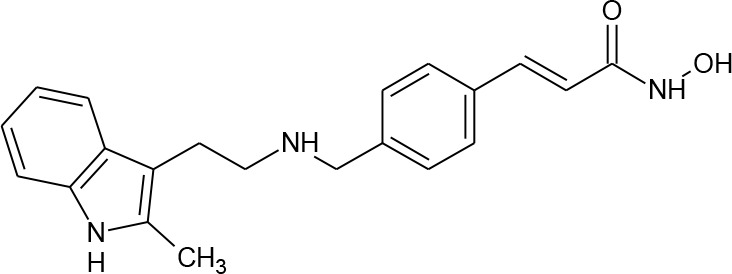	6918837	Rasmussen et al., 2014	640
8	Psammaplin A*	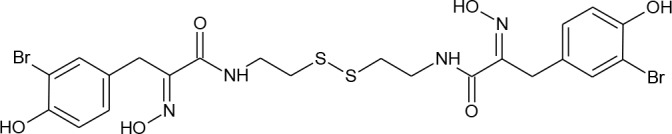	6400741	Richard et al., 2018	77
9	Romidepsin*	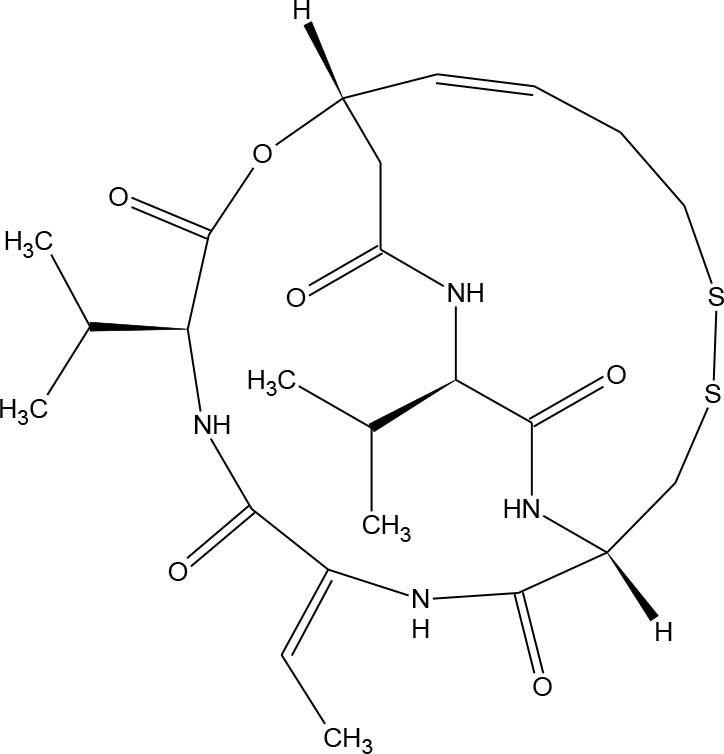	5352062	Wei et al., 2014	244
10	Scriptaid	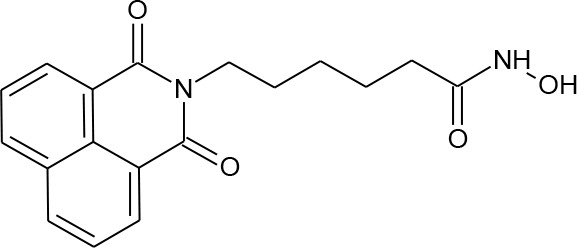	5186	Ying et al., 2010	554
11	Serpulanine A*	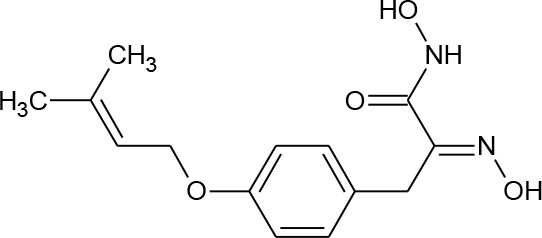	N/A	Williams et al., 2018	29
12	Trichostatin A*	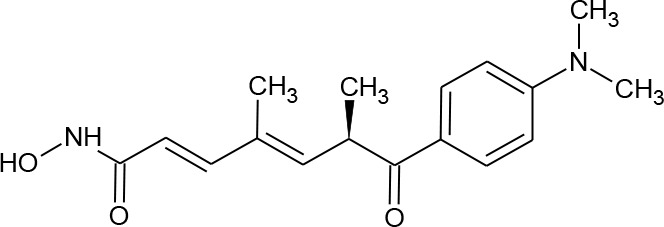	444732	Kiernan et al., 1999	355
13	Valproic acid	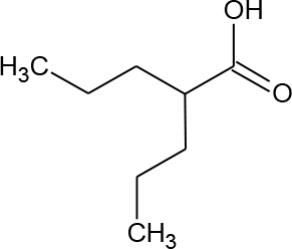	3121	Matalon et al., 2011	403
14	Vorinostat	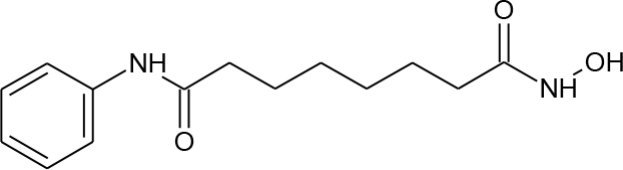	5311	Contreras et al., 2009; Archin et al., 2012	340

*indicates natural product.

### Protein Preparation

The high-resolution crystal structure of the human histone deacetylase 1 (hmHDAC1, class I HDAC family), with PDB ID: 5ICN, chain B, (Resolution: 3.3 Å) was downloaded from the Protein Databank (PDB; www.rcsb.org; Burley et al., 2018). Preparation of the protein structure was performed using protocols similar to what we have previously reported (Heimburg et al., 2016; Simoben et al., 2018). MOE software (v. 2016.08) was used to delete all water molecules. Further preparation steps on the protein were applied using the default settings of the Protein Preparation Wizard of Schrödinger software (Sastry et al., 2013). Assignment of the bond orders and hydrogen atoms as well as protonation of the heteroatom states were added using Epik-tool (with the pH set at biologically relevant values, i.e., at 7.0 ± 2.0). Optimization of the H-bond network was done, and the structure was finally subjected to a restrained energy minimization step using the Optimized Potentials for Liquid Simulations (OPLS) 2005 force field. The root-mean-square deviation (RMSD) of the atom displacement for terminating the minimization was 0.3 Å (Banks et al., 2005).

### Ligand Dataset Preparation

Ligands for docking were prepared using similarly reported protocols (Heimburg et al., 2016; Simoben et al., 2018). Preparation of ligands for docking was done using the LigPrep tool (Schroedinger, 2017-u1), as implemented in Schrödinger's software (version 2017-1). All possible tautomers, as well as possible combinations of stereoisomers for molecules without well-defined stereochemistries, were generated for pH 7.0 ± 2.0 using the Epik ionization method. Additionally, the optimized integrated OPLS-2005 force field (Banks et al., 2005) was used to minimize all ligands. Pan-Assay Interference (PAIN) molecules were subsequently discarded after the application of PAIN filters implemented in the Schroedinger's Canvas tool. Finally, the generation of 30 conformers for each of the prepared ligand molecules, followed by the minimization of each conformer output, was performed using the settings of ConfGen (Watts et al., 2010).

### Ligand Docking and Scoring

Prepared protein and ligands were docked using the standardized Glide program procedure of Schrödinger's software (version 2017-1; Friesner et al., 2004; Halgren et al., 2004). The receptor grid preparation for the docking procedure was carried out by assigning the nonpolymer fraction of the cocrystallized ligand (6A0) as the centroid of the grid box. The generated three-dimensional conformers of the prepared ligand were subsequently docked into the receptor model using the Glide program. An additional metal constraint on the catalytic Zn^2+^ ion was used to optimize our docking results. A total of five docking poses per ligand conformer were included in the postdocking minimization step, and a maximum of two docking poses was generated for each ligand conformer. The GlideScore Standard Precision (SP) score mode was used as the scoring function to rank the resulting binding poses (Friesner et al., 2004; Halgren et al., 2004). The Schroedinger clustering package was subsequently used to cluster the resulting poses using an RMSD value of 1.5 Å and the Glide SP score as properties of interest. Further reduction in the number of molecules was done by choosing only the top 200,000 docking poses each from the clustered outcomes after ranking by Glide SP score and Glide Ligand efficiency (LE) scores. The resulting sets of molecules were merged and duplicates were removed. The final set of hits was proposed after manual inspection of each docking pose and interaction(s) with the conserved amino acid residues His140, His141, and Tyr303 in the HDAC active site.

### HDAC Inhibition Assays

HDAC activity in the presence of compounds was performed using the HDAC-Glo I/II assay (Promega) according to the manufacturer's instructions. HDAC reactions were performed in white 384-well plates with a final volume of 20 µl per well. Stock compounds and DMSO were diluted in manufacturer buffer to desired concentrations and added to wells. Jurkat cells were then resuspended in phenol red-free and fetal bovine serum-free RPMI 1640 and seeded into wells at 3*10^3^ cells/well. Wells containing no cells were included as negative controls for the signal background. Following incubation at 37°C and 5% CO_2_ for 90 min, 20 µl of HDAC-Glo I/II Reagent plus 1% Triton-X100 (prepared as per manufacturer's instructions) was added to each well. Plates were gently mixed for 30 s and then incubated at room temperature for an additional 30 min. Luminescence was detected using an Infinity M200 multimode plate reader (Tecan Life Sciences). Data were normalized between no-inhibitor (100%) and no-cell (0%) controls and presented as the mean ± s.e.m. from three independent experiments.

### *In Vitro* HIV Latency Reversal Assays

J-Lat 10.6 cells were seeded in 96-well plates at 2*10^5^ cells/well and coincubated with LRAs at defined concentrations or 0.1 to 1.0% DMSO vehicle control for 24 or 72 h. Cells were then examined for GFP expression by flow cytometry (Guava EasyCyte 8HT, Millipore). Culture viability was estimated by flow cytometry and based on the relative percentage of compound-treated J-Lat cells displaying the characteristic forward- and side-scatter parameters of vehicle-treated control cells (Tietjen et al., 2015). Flow cytometry data were analyzed using FlowJo v. 10.5.3 software (FlowJo LLC, Ashland, OR), where background GFP signals in live J-Lat cells treated with respective DMSO concentrations were set to 0.05%-positive cells. For all results, data are presented as the mean ± s.e.m. from at least three independent experiments.

## Results

### Virtual Screening to Identify Putative HDAC Inhibitors and LRAs

**Table 1** lists the structures of 14 compounds (compounds **1–14**) that were used for similarity searching in PubChem. **Table 2** lists published activities of these 14 compounds to inhibit recombinant HDAC1. All selected compounds were also reported to reverse HIV latency in one or more *in vitro* and/or primary cell assays with the exception of serpulanine A (Williams et al., 2018), which we confirmed at 10 µM induced a 31.0% increase in HIV provirus expression, as measured by the GFP reporter, in J-Lat 10.6 cells (Jordan et al., 2003 and see below). A similarity search was then performed to reduce the virtual chemical space to a manageable number of molecules for VS (see Materials and Methods), which resulted in 6,175 compounds. After the removal of duplicates and pan assay interference molecules, we obtained 5,867 molecules for VS.

**Table 2:** Reported inhibition of recombinant HDAC1 by histone deacetylase (HDAC) inhibitors.

Number	Compound	Reference/PubChem BioAssay ID Measure (µM)							
		Wagner et al., 2016	Schultz et al., 2011	Carrillo et al., 2015	Bradner et al., 2010	Phiel et al., 2001; Huber et al., 2011	Baud et al., 2012	Vickers et al., 2012	Williams et al., 2018
		1313935	609489	1236442	496801	N/A	670013	673990	N/A
		IC_50_	IC_50_	K_i_	K_i_	IC_50_	IC_50_	IC_50_	IC_50_
1	TPB	0.0002							
2	Belinostat		0.015	0.0009	0.00085				
3	Etinostat		0.485	0.002	0.022				
4	Givinostat				0.002				
5	Mocetinostat		0.152		0.009				
6	Oxamflatin					0.003959			
7	Panobinostat	0.001		0.001	0.001				
8	Psammaplin A*						0.045		
9	Romidepsin*							0.0002	
10	Scriptaid				0.0015	0.006421			
11	Serpulanine A*								7.2
12	Trichostatin A*			0.0002	0.0002	0.0169			0.5
13	Valproic acid					171 (Huber et al., 2011); 400 (Phiel et al., 2001)			
14	Vorinostat		0.077		0.0013	0.0137	0.03	0.069	

*indicates natural product.

**Figure 1:**
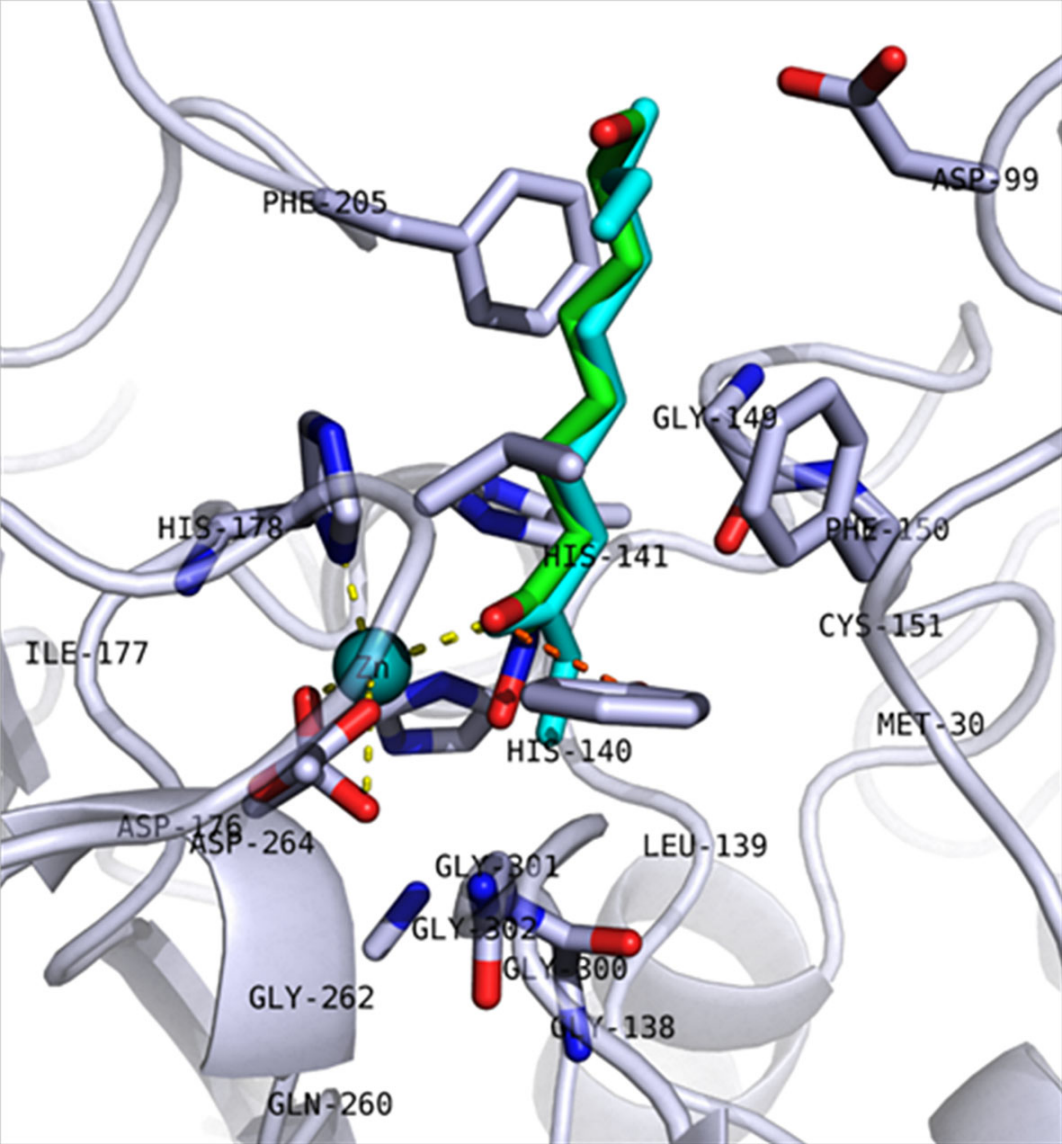
Redocking pose of 5ICN_B native ligand (6A0). The backbone of the protein is shown as a cartoon, and key amino acid residues in the active site are shown in stick representation. The redocked molecule is shown in cyan, while the crystalized pose is shown in green. Coordination of Zn^2+^ ion (cyan sphere) and hydrogen bond interactions with Tyr303 are shown as yellow and orange dashed lines, respectively.

**Figure 2:**
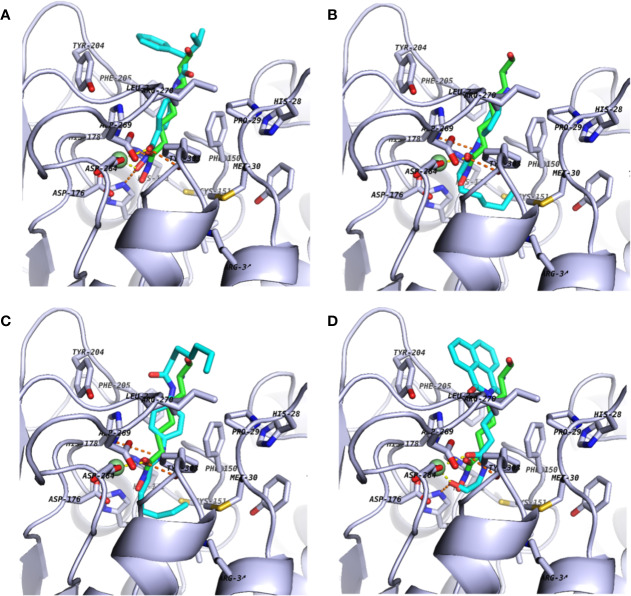
Docking poses of commercially available screening hits: **(A)** (S)-*N*-Hydroxy-4-(3-methyl-2-phenylbutanamido)benzamide (compound **15**); **(B)** *N*-(4-Aminophenyl)heptanamide **(16)**; (**C**) *N*-[4-(Heptanoylamino)phenyl]heptanamide (**17**); and **(D)** 4-(1,3-Dioxo-1H-benzo[de]isoquinolin-2(3H)-yl)-*N*-(2-hydroxyethyl)butanamide **(18)**. Schematics are shown as described in **Figure 1**.

These 5,867 molecules were next docked into the active site of the hmHDAC1 protein (PDB ID: 5ICN, chain B; Resolution: 3.3 Å). The proposed docking procedure was able to redock the cocrystallized inhibitor within the receptor-binding pocket with RMSD < 1 Å. The redocked inhibitor pose was observed to reproduce the bidentate coordination to the catalytic Zn-ion as well as H-bond interaction with the conserved Tyr303 (**Figure 1**). Further reduction of the compound set was then performed *via* clustering of the docking results (generated after sorting by docking score as well as ligand efficiency) using an average RMSD of 1.5 Å base on the docking score. The final selection and proposal of hits were based on visual inspection of the selected docked poses for conserved interactions (such as coordination to the conserved catalytic Zn^2+^ ion and hydrogen bond interactions) and based on published works (**Figure 2**).

**Figure 3:**
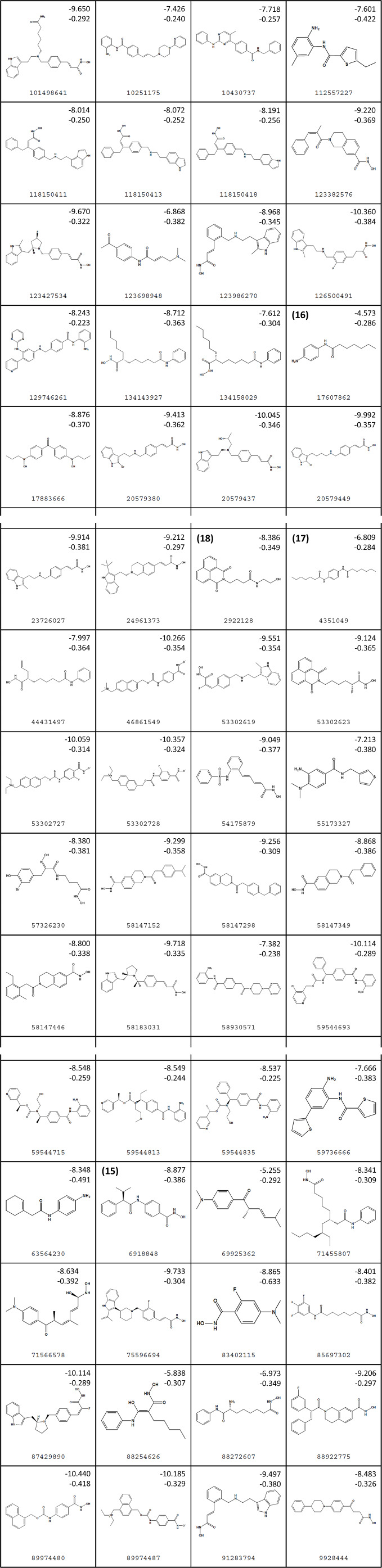
Structures of virtual screening (VS) hits. Compounds **15–18** are highlighted. For each structure, PubChem ID numbers are located at bottom center. Values at top right indicate Glide Standard Precision (SP) (top) and Glide Ligand efficiency (LE) (bottom) scores.

**Figure 3** lists 60 compounds that were identified as putative hits of the VS. From these 60 compounds, four (6.6%) including (S)-*N*-Hydroxy-4-(3-methyl-2-phenylbutanamido)benzamide (compound **15**), *N*-(4-Aminophenyl)heptanamide (**16**), *N*-[4-(Heptanoylamino)phenyl]heptanamide (**17**), and 4-(1,3-Dioxo-1H-benzo[de]isoquinolin-2(3H)-yl)-*N*-(2-hydroxyethyl)butanamide (**18**), were readily available through commercial sources and therefore selected for further validation in functional studies (**Table 3**). Compound **15** contains a hydroxamic acid and the same core pharmacophore as compound **1**. Also called AR-42, **15** was previously reported to reverse HIV latency *in vitro* (Mates et al., 2015) but was not included in our original list of HDAC inhibitors for VS, providing initial validation of this experimental approach. Compounds **16-17** are both *N*-phenylheptamides, with **17** almost being the dimeric form of **16** (except for an additional amine group). **16** and **17** structurally resemble vorinostat (**14**), however missing the crucial hydroxamic acid moiety. Finally, compound **18** resembles scriptaid (compound **10**; Ying et al., 2010) with the difference being that **18** has an amide and a hydroxyl group, separated by two methylene groups, in place of the Zinc-binding hydroxamic acid moiety in scriptaid and which is often found in HDAC inhibitors. The abilities of **16–18** to inhibit HDAC and reverse HIV latency have not been reported.

**Table 3:** Compounds hits from virtual screening (VS) selected for biological validation.

Compound		Structure	Reported HIV latency reversal?
15	(S)-*N*-Hydroxy-4-(3-methyl-2-phenylbutanamido)benzamide	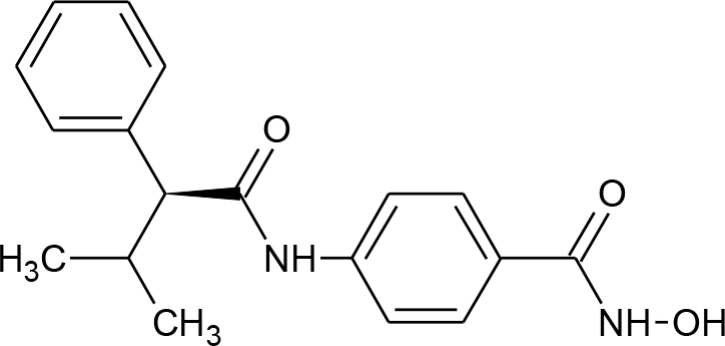	Mates et al., 2015
16	*N*-(4-Aminophenyl)heptanamide	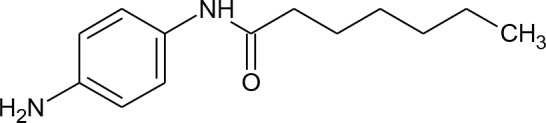	no
17	*N*-[4-(Heptanoylamino)phenyl]heptanamide	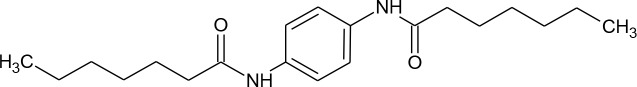	no
18	4-(1,3-Dioxo-1H-benzo[de]isoquinolin-2(3H)-yl)-*N*-(2-hydroxyethyl)butanamide	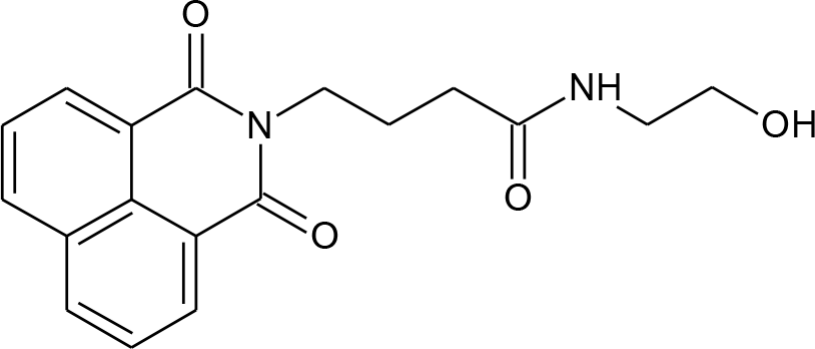	no

### Effects of VS Hits on HDAC Inhibition

To examine whether compounds **15–18** inhibit HDAC activity *in vitro*, we used the HDAC-Glo I/II assay (Promega). This assay allows quantification of HDAC I/II activity in live cells *via* a cell-permeable, acetylated, luminogenic peptide substrate. The deacetylated peptide is then cleaved by the addition of a developer reagent, which releases aminoluciferin from the peptide. Finally, aminoluciferin release is quantified in a firefly luciferase reaction. As a result, increased deacetylation of the substrate by cellular HDACs results in increased firefly luciferase production, while suppression of HDAC activity with inhibitors results in decreased luminescence production.

**Figure 4:**
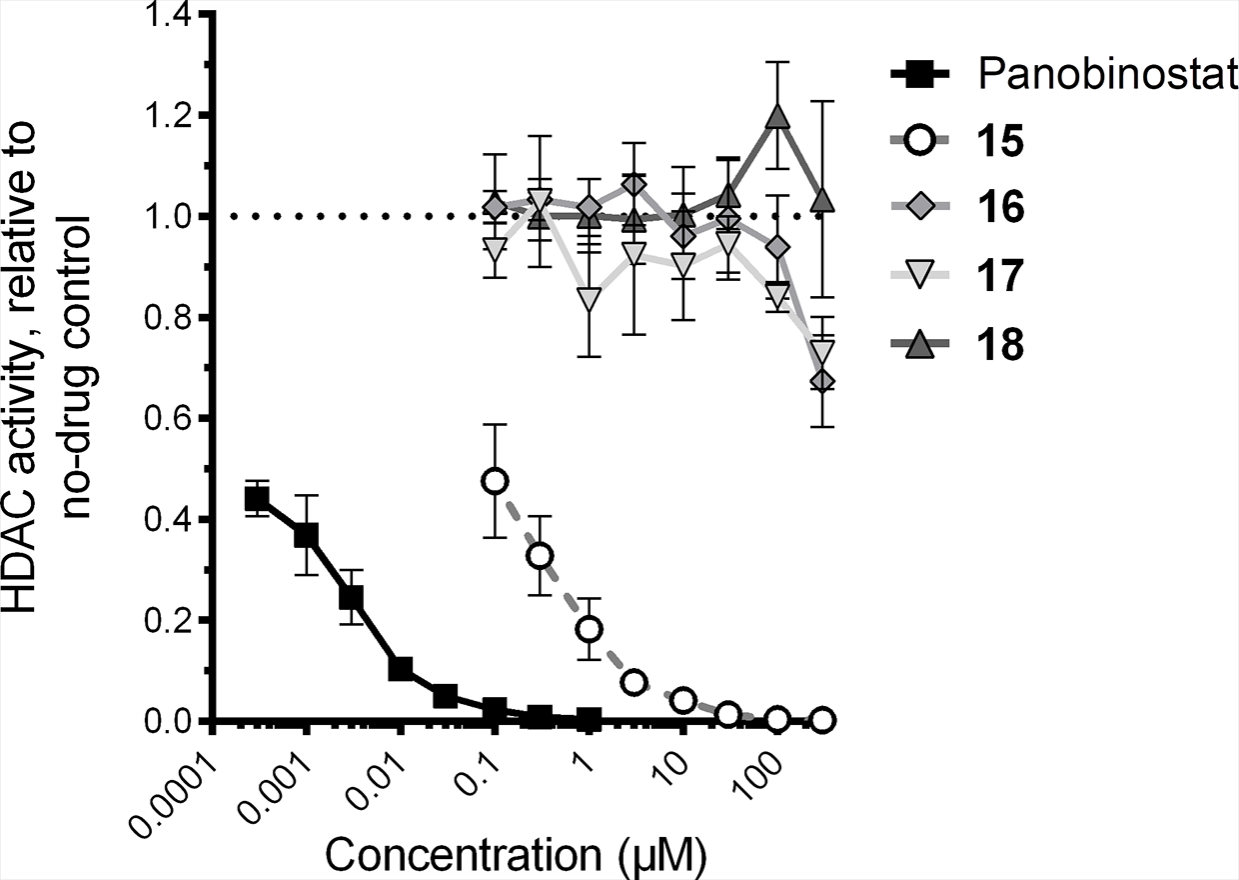
Effects of four virtual screening (VS) hits on cellular histone deacetylase (HDAC) activity, as recorded by HDAC-Glo assay. HDAC activity in the presence of each compound is presented relative to HDAC activity in cells treated with 0.1% DMSO vehicle control.

Using this assay, the treatment of Jurkat cells with the control HDAC inhibitor panobinostat (compound **7**) resulted, as expected, in substantial inhibition of *in vitro* HDAC activity (**Figure 4**). For example, as little as 300 pM of panobinostat inhibited 55.9% ± 3.5% of HDAC activity (mean ± s.e.m.), with > 99% HDAC inhibition observed at 0.3 µM, which is consistent with other results from *in vitro* assays (Scuto et al., 2008). Also consistent with its known activity as an HDAC inhibitor (Mates et al., 2015), compound **15** additionally inhibited HDAC activity, albeit at much higher concentrations (e.g., 52.5% ± 11.2% inhibition at 0.1 µM and > 99% inhibition at 100 µM; **Figure 4**). In contrast, no more than 32.7% ± 9.1% and 27.1% ± 7.2% inhibition at 300 µM were observed for compounds **16** and **17**, respectively, indicating weak but detectable HDAC inhibition *in vitro*. No inhibitory activity was detected for compound **18** at any concentration up to 300 µM. Thus three of the four assessed VS hits detectibly inhibit HDAC activity in Jurkat cells, although none approach the activity of the control HDAC inhibitor panobinostat.

### Effects of VS Hits on *In Vitro* HIV Latency Reversal

**Figure 5:**
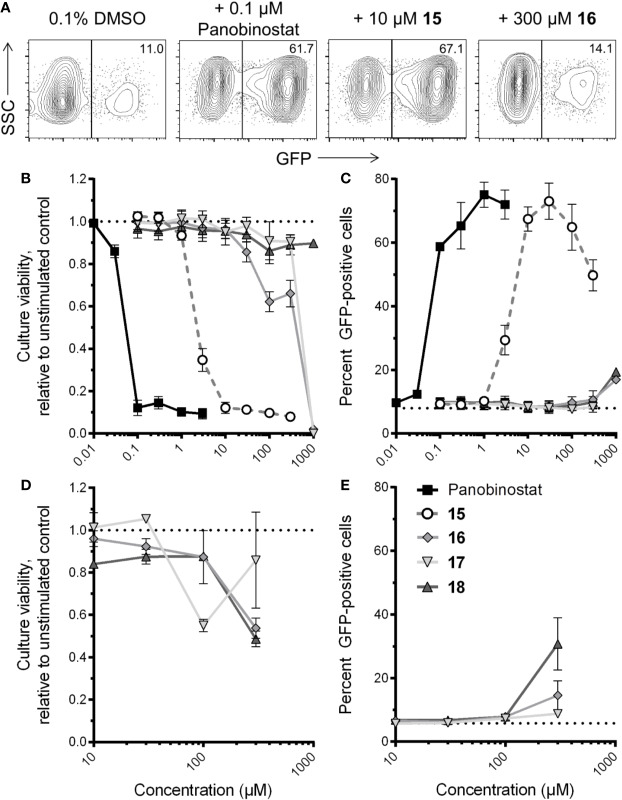
Effects of four virtual screening (VS) compound hits on HIV latency reversal. **(A)** Representative flow cytometry data of HIV latency reversal, as measured by GFP expression, in J-Lat 10.6 cells in the presence of 0.1% DMSO, panobinostat, or select VS hits following 24-h incubation. For each panel, values indicate percent GFP-positive cells. **(B)** Dose-response profiles of panobinostat and compounds **15–18** on J-Lat 10.6 estimated cell culture viability following 24-h incubation. Data are presented relative to culture viability of cells treated with 0.1% DMSO vehicle control. **(C)** Dose-response profiles of compounds on HIV-1 latency reversal, as measured by percent GFP-positive cells, following 24-h incubation. Dotted line indicates baseline GFP expression in untreated cells. **(D)** Effects of compounds **16–18** on J-Lat 10.6 estimated culture viability after 72-h incubation. Data are presented as described in **(B)**. **(E)** Effects of **16–18** on HIV-1 latency reversal after 72-h incubation. Data are presented as described in **(C)**.

To investigate whether compounds **15–18** reverse HIV latency *in vitro*, we used the J-Lat 10.6 cell line. These cells are derived from Jurkat T cells and contain an inducible latent provirus with a frameshift mutation in Env, rendering the virus noninfectious, in addition to a GFP reporter expressed from the deleted viral Nef locus (Jordan et al., 2003). As such, HIV latency reversal in these cells can be measured by an increase in GFP expression, as measured by flow cytometry. Resting J-Lat 10.6 cells feature a proportion of spontaneously GFP-expressing cells that are not fully latent; however, they are also highly sensitive to stimulation by LRAs (Williams et al., 2004; Cummins et al., 2017). In our hands, we observed an average of 8.0% ± 1.7% GFP-positive cells in resting conditions. J-Lat 10.6 cells were therefore treated with panobinostat, compounds **15–18**, or DMSO vehicle control for 24 h. **Figure 5A** shows representative examples of GFP expression in J-Lat 10.6 cells plus LRAs.

In parallel, we also estimated cell culture viability by measuring the percentage of LRA-treated cells displaying the characteristic forward and side-scatter parameters of control cultures treated with 0.1% DMSO (Tietjen et al., 2015; **Figure 5B**). After 24-h incubation, the control HDAC inhibitor panobinostat was observed to induce substantial cytotoxicity, consistent with our previous observations (Richard et al., 2018): for example, treatment with 0.1 µM panobinostat resulted in an estimated 12.1% ± 3.6% culture viability relative to cells treated with 0.1% DMSO, and a 50% cytotoxicity concentration (CC_50_) of 35.3 ± 4.1 nM (**Figure 5B**). Compound **15** exhibited a similar level of toxicity at 30 µM (11.3% ± 2.0% estimated culture viability), with a CC_50_ of 1.6 ± 0.3 µM, thereby indicating a comparable therapeutic window to panobinostat in this assay. Substantial cellular toxicity (i.e. > 50%) for compounds **16** and **17** were not observed except at 1 mM, where estimated culture viabilities for both relative to DMSO-treated cells were 2.0% ± 0.0% and 0.0% ± 0.0%, respectively, while no substantial toxicity was observed for compound **18** at any concentration up to 1 mM (i.e. minimum 86.1% ± 6.0% estimated culture viability at 100 µM; **Figure 5B**). Thus, when panobinostat and compounds **15–18** are rank-ordered, these activities match their respective *in vitro* HDAC inhibition efficacies (**Figure 4**).

Most compounds were also observed to reverse HIV latency in live cells after 24 h, as measured by GFP expression (**Figure 5C**). For example, in the presence of panobinostat, GFP was detected in a maximum of 75.1% ± 3.9% of cells at 1 µM, while a maximum of 73.0% ± 5.7% of cells were GFP-positive in the presence of 30 µM of compound **15**. In contrast, no more than 16.9% ± 0.2% GFP expression in live cells was observed in the presence of compound **16**, which occurred at 1 mM, reflecting only a 2.1-fold induction of GFP over spontaneous expression in DMSO-treated cells (**Figure 5C**). For compound **17**, no GFP expression was observed at any concentration, although no data could be obtained at 1 mM due to complete cytotoxicity. Interestingly, despite exhibiting no *in vitro* HDAC inhibition or toxicity, compound **18** did induce weak but consistent GFP expression in 19.4% ± 0.2% of cells at 1 mM, or 2.4-fold over spontaneous expression. These results indicate that compound **15** is a robust LRA, although with efficacy approximately one to two orders of magnitude lower than control panobinostat, while compounds **18** and **16** may exhibit latency reversal at very high concentrations.

To confirm whether compounds **16–18** could consistently induce latency reversal at levels above spontaneous GFP expression in DMSO-treated J-Lat 10.6 cells, we repeated the assays described above but incubated cells for a total of 72 h. In these conditions, no more than 50% loss of estimated culture viability was observed for any compound at up to 300 µM. (**Figure 5D**). Notably, at 300 µM, compounds **16** and **17** consistently induced 15.5% ± 4.6% and 8.8% ± 1.4% at 300 µM, respectively, or 2.7 and 1.5-fold increased GFP over spontaneous expression (5.8% ± 0.2%; **Figure 5E**). Interestingly, 300 µM of compound **18** induced 30.8% ± 8.2% GFP expression, a 5.3-fold increase over spontaneous expression, indicating consistent HIV latency reversal despite no activity in HDAC inhibition assays and no obvious cytotoxicity.

Taken together, these results indicate that compounds **15–18** all induce latency reversal and/or cytotoxicity, albeit at concentrations that are orders of magnitude higher than panobinostat.

## Discussion

Current LRAs used in shock-and-kill therapeutic strategies to date have yet to consistently clear viral reservoirs in humans, prompting the need to identify new chemical leads to inform ongoing efforts to improve existing LRA strategies. Toward this goal, we described here a VS-based approach to probe 5,867 nonredundant molecules from PubChem to identify 60 which structurally resemble LRAs of the HDAC inhibitor functional class. The four compounds that were readily commercially available were further tested and confirmed to inhibit HDAC activity and/or reverse HIV latency in vitro. In a related approach, Gallastegui et al. (2012) used the J-Lat A2 cell line to screen 6,000 small molecules in vitro, and a VS-based approach was then used to probe 7.5 million compounds to identify those with similarity to the eight biologically identified hits. This strategy led to discovery of the novel LRA 8-methoxy-6-methylquinolin-4-ol (Gallastegui et al., 2012). Our study, which used VS to discover new LRAs of the HDAC inhibitor class, further demonstrates that new LRAs can be readily identified using similarity-based VS approachesWhile none of the compounds reached the efficacy of the control HDAC inhibitor panobinostat in our study, this we demonstrate that this proof-of-concept approach represents a simple, efficient, and cost-effective method to identify new LRA leads. The VS approaches described here are readily transferrable to resource-constrained researchers and also allow improved access and ability toward informing worldwide HIV eradication efforts for even remote and very small research groups. However, while VS can be faster and more cost-effective than biological laboratory-based compound screens, care must be taken as several methods for scoring active and inactive compounds are often unsuccessful (Chen, 2015). Another caveat is compound availability for biological validation; for example, a recent study showed that only ~10% of compounds contained in electronic libraries potentially identifiable by VS are accessible as samples through commercial sources and academic collaborations (Chen et al., 2017). This clearly impacted our ability to validate all hits identified in the VS, as only four compounds (6.7%) were available to us from commercial sources. The low commercial availability of identified hits is therefore a significant limitation of this study and precludes determining statistically whether identifying hits by VS is likely to translate into functional effects. However, the published compounds available in PubChem, even if not commercially available, can still inform further design studies such as chemical optimization from a starting point. Future synthetic chemistry efforts beyond the scope of this work will likely be required to address this question. Limited availability becomes even more heightened when rare compounds such as those obtained in natural product-based isolation efforts are considered, and this highlights the continuing need for the building and efficient management and accessibility of consortiums of publicly available synthetic and natural compound repositories (Ntie-Kang et al., 2014; Tietjen et al., 2015; Ntie-Kang et al., 2017). Furthermore, as biological activities for compounds **16–8** were only found at concentrations > 100 µM, synthetic chemistry efforts are needed to identify derivatives with improved efficacies before additional studies are pursued, for example to assess ability to reverse viral latency in primary cells from donors with HIV. Future VS efforts should also focus on identifying compounds with structural similarities to only the most efficacious HDAC inhibitors.

Of the four VS hits validated here, (S)-N-Hydroxy-4-(3-methyl-2-phenylbutanamido)benzamide (15), also called AR-42, was previously described as an HDAC inhibitor and HIV LRA with cell toxicity at single micromolar to high nanomolar concentrations (Mates et al., 2015). Our results are consistent with this previous study. As 15 was not included in our initial set of screening probes, the rediscovery of 15 here supports both the previously reported results of Mates et al. but also serves as a positive control for the VS approach described here. In contrast, compounds 16 and 17 exhibited limited HDAC1 inhibition at 300 µM (32.7 ± 9.1 and 27.1% ± 7.2% respectively) which, although clearly weak functional hits, do place them within the range of efficacies observed for the established HDAC inhibitor valproic acid in similarly designed assays (i.e., reported IC_50_s = 171–400 µM; **Table 2**; Phiel et al., 2001; Huber et al., 2011). Furthermore, while 16 and 17 respectively induced only 2.7 or 1.5-fold increased GFP over spontaneous expression at 300 µM in J-Lat 10.6 cells, these results are consistent with the ability of valproic acid to induce latency reversal in the comparable Jurkat-LAT-GFP cell line, where 1 and 2.5 mM resulted in 1.6- and 2.2-fold increases in GFP expression, respectively (Pérez et al., 2010). Notably, 4-(1,3-Dioxo-1H-benzo[de]isoquinolin-2(3H)-yl)-*N*-(2-hydroxyethyl)butanamide (compound **18**) was also able to weakly reverse HIV latency at high concentrations (> 100 µM) but did not inhibit *in vitro* HDAC activity at up to 300 µM. This result was unexpected from a biological perspective, although not from a chemical perspective as **18** has no zinc-chelating moiety. The reason for the discrepancy in biological assays for **18** is not immediately clear but could reflect exceptionally slow binding of HDACs *in vitro* which is not captured within the time-course of the HDAC-Glo assay performed here. However, the parent compound of **18** (scriptaid) has also been reported to act on other biological pathways which drive HIV latency reversal including inhibition of histone methyltransferase at H3K9 moieties, induction of PI3K/AKT signaling, and perhaps other putative activities which allow scriptaid to synergize with other HDAC inhibitors *in vitro* (Chen et al., 2013; Wang et al., 2013; Liang et al., 2015; Andersen et al., 2018). Thus **18** could function as an LRA through one of these alternative proviral signaling pathways. However, further studies with more potent analogs of **18** and detailing of structure-activity relations based on the scriptaid scaffold are desired to test this hypothesis.

In summary, we demonstrate that VS-based approaches can readily identify novel LRA candidates of the HDAC inhibitor functional class. This approach can be used, in principle, to identify additional, novel LRAs representing other functional classes such as PKC activators, BET bromodomain inhibitors, and others (Andersen et al., 2018). It also identifies new chemical scaffolds which can serve as starting points to design more potent LRAs and which may be useful toward improving shock-and-kill based HIV eradication therapies in humans.

## Data Availability Statement

All datasets generated for this study are included in the article/supplementary material.

## Author Contributions

DD, CVS, CS, and KR performed experiments and wrote and reviewed the manuscript. WS, FN-K, and IT conceived the study, planned and supervised experiments, and wrote the manuscript. All authors contributed to the article and approved the submitted version.

## Funding

Funding was provided by the Canadian Institutes for Health Research (CIHR PJT-153057) (IT) and the New Frontiers in Research Fund – Explorations (NFRFE-2018-01386) (IT). This work was also supported through the Sub-Saharan African Network for TB/HIV Research Excellence (SANTHE) (KR, IT), a DELTAS African Initiative [grant no. DEL-15-006]. The DELTAS Africa Initiative is an independent funding scheme of the African Academy of Sciences (AAS)'s Alliance for Accelerating Excellence in Science in Africa (AESA) and supported by the New Partnership for Africa's Development Planning and Coordinating Agency (NEPAD Agency) with funding from the Wellcome Trust [grant no. 107752/Z/15/Z] and the UK government. DD was supported by a Simon Fraser University – Social Sciences and Humanities Research Council of Canada (SSHRC) Undergraduate Research Student Award. CVS acknowledges financial support from the German Academic Exchange Services (DAAD), Germany, awarded to Doctoral Candidates. CS was supported by a CIHR Frederick Banting and Charles Best MSc Award. KR was a recipient of a Canadian Queen Elizabeth II Diamond Jubilee Scholarship, a partnership between the Rideau Hall Foundation, Community Foundations of Canada and Universities Canada, in addition to a SANTHE Ph.D. Fellowship. FN-K acknowledges a return fellowship and equipment subsidy from the Alexander von Humboldt Foundation, Germany.

## Conflict of Interest

The authors declare that the research was conducted in the absence of any commercial or financial relationships that could be construed as a potential conflict of interest.

## Abbreviations

cART, combination antiretroviral therapy; CC_50_, 50% cytotoxicity concentration; HDAC, histone deacetylase; LE, ligand efficiency; LRA, latency reversal agent; OPLS, optimized potentials for liquid simulations; PAIN, pan-assay interference; PDB, protein data bank; RMSD, root-mean-square deviation; SP, standard precision; VS, virtual screening; 2D, two dimensional.
